# Endothelial Cells Provide a Notch-Dependent Pro-Tumoral Niche for Enhancing Breast Cancer Survival, Stemness and Pro-Metastatic Properties

**DOI:** 10.1371/journal.pone.0112424

**Published:** 2014-11-07

**Authors:** Pegah Ghiabi, Jie Jiang, Jennifer Pasquier, Mahtab Maleki, Nadine Abu-Kaoud, Shahin Rafii, Arash Rafii

**Affiliations:** 1 Stem Cell and Microenvironment Laboratory, Weill Cornell Medical College in Qatar, Education City, Qatar Foundation, Doha, Qatar; 2 Department of Genetic Medicine, Weill Cornell Medical College, New York, New York, United States of America; Mayo Clinic College of Medicine, United States of America

## Abstract

Treating metastasis has been challenging due to tumors complexity and heterogeneity. This complexity is partly related to the crosstalk between tumor and its microenvironment. Endothelial cells -the building blocks of tumor vasculature- have been shown to have additional roles in cancer progression than angiogenesis and supplying oxygen and nutrients. Here, we show an alternative role for endothelial cells in supporting breast cancer growth and spreading independent of their vascular functions. Using endothelial cells and breast cancer cell lines MDA-MB231 and MCF-7, we developed co-culture systems to study the influence of tumor endothelium on breast tumor development by both *in vitro* and *in vivo* approaches. Our results demonstrated that endothelial cells conferred survival advantage to tumor cells under complete starvation and enriched the CD44^High^CD24^Low/-^ stem cell population in tumor cells. Moreover, endothelial cells enhanced the pro-metastatic potential of breast cancer cells. The *in vitro* and *in vivo* results concordantly confirmed a role for endothelial Jagged1 to promote breast tumor through notch activation. Here, we propose a role for endothelial cells in enhancing breast cancer progression, stemness, and pro-metastatic traits through a perfusion-independent manner. Our findings may be beneficial in developing novel therapeutic approaches.

## Introduction

Breast cancer is the most commonly diagnosed cancer and the second cause of mortality in women in the western world [1]. Most breast cancer patients die due to tumor metastasis. Preventing breast cancer recurrence and metastasis seems challenging owing to disease complexity. In addition to tumor heterogeneity, this complexity can be in part attributed to the interaction between tumor cells and their microenvironment. The components of tumor microenvironment comprise of epithelial, endothelial, bone-marrow mesenchymal, and immune cells, as well as the elements of the extracellular matrix. The crosstalk between tumor cells and their surrounding microenvironment seems to be crucial for tumor growth, development, stemness, and metastatic spread [2].

Endothelial cells (ECs) constitute the main building blocks of blood vessels and are responsible for tumor angiogenesis, which greatly influence tumor progression and spreading [3]–[5]. However, the relative failure of anti-angiogenic therapies despite vessel disruption illustrates the existence of an alternative function for ECs and proposes a more complex role for the vascular network in tumor development. In recent years, it has been shown that the tumor ECs release specific growth factors called angiocrine factors, which might directly regulate tumor growth in a perfusion-independent manner [6]–[10]. There is evidence on involvement of several angiocrine factors in organogenesis, which indicates their potential ability to influence tumor growth in adulthood [11]–[13]. Recent reports have shown the participation of ECs in growth and maintenance of several cancer types [10], [14]–[17]. However, the intracellular signaling pathways that mediate tumor-endothelial interaction need further validation. Notch signaling is implicated in normal mammary development, promotion of tumor malignancy, maintenance of cancer stem cells, and development of tumor pro-metastatic phenotype [18], [19]. In addition, notch is reportedly involved in tumor angiogenesis through interaction with surrounding vasculature [20]–[22]. Therefore, a role for Notch pathway in regulation of tumor-endothelial crosstalk should be considered.

In this study, we aimed at investigating the interaction of breast cancer cells (BCCs) MDA-MB231 and MCF-7 with ECs in a co-culture system. In order to minimize the background effect of serum and cytokines on BCC/ECs interaction, we performed all the experiments under starvation condition. To overcome the hurdle of rapid cell death while starving primary ECs *in vitro*, we utilized a genetically modified form of primary ECs (PECs) transfected with adenoviral *E4ORF1* gene as described previously to obtain E4-ECs [23]. While this transfection provides a low Akt activation allowing E4-ECs survival in a serum and cytokine-free condition, it does not modify their endothelial phenotype as we have previously reported [10], [24], [25]. Besides, activation of Akt in tumor endothelium has been previously reported [26] and our model might thus be more optimal to mimic the crosstalk between ECs and cancer cells *in vivo*. We demonstrated that E4-ECs are capable of supporting breast cancer growth, stemness, and invasiveness. Moreover, we provide strong evidence that E4-EC induction of breast tumor Notch pathway plays a role in this crosstalk since its inhibition significantly weakened the endothelial effect on tumor growth and stemness *in vivo*.

## Material and Methods

### Cell proliferation assay

Breast cancer cell lines MDA-MB231 (MDA-231) and MCF-7 were purchased from American Type Culture Collection (ATCC). Breast cancer cells (BCCs) were grown in DMEM/High glucose (Sigma, USA) supplemented with 10% FBS, L-glutamine, non-essential amino acids (NEAA), and penicillin/streptomycin in a humidified incubator with 5% CO_2_. Gamma secretase inhibitor (GSI) was a product of Sigma, USA. E4-ECs were obtained as described earlier [23]. E4-ECs were cultivated in M199 media (Sigma, USA) supplemented with 20% FBS, 20 µg/ml β-Endothelial Cell Growth Factor (βECG), and 20 units/ml heparin. Cell culture inserts (transwells) were purchased from BD Biosciences (USA). BCCs and GFP^+^E4-ECs or mCherryE4-ECs were co-cultivated at 1∶5 ratio for 2, 4, and 7 days devoid of serum and cytokine and proliferation and survival of BCCs were assessed by counting GFP^-^BCCs under AMG EVOS fluorescent microscope. GSI was added to the co-cultures on a daily basis at a concentration of 5 µM to inhibit notch pathway.

### Sphere forming assay

PKH26 fluorescent cell linker (PE-conjugated) dye was purchased from Sigma (USA) and used according to the manufacturer's instruction. BCCs were initially stained with PKH26 dye and enriched as mammospheres in so-called *3D media* under non-adherent condition in ultralow attachment plates (Corning, USA) following the method previously described by Dontu et al. [27]. The *3D* media was made of DMEM-F12 (Sigma, USA) supplemented with 2% B27, 5 µg/mL insulin, 20 ng/mL basic fibroblast growth factor (bFGF) and epidermal growth factor (EGF). In order to prevent the formation of cellular aggregates, a highly viscose 3D media was prepared by the addition of 0.2% methylcellulose to the above mixture (Sigma, USA). To make mammospheres, PKH26^+^BCCs were seeded at 10^3^−5×10^3^ cells/mL of 3D media and cultured for 5–7 days to obtain primary mammospheres. Primary mammospheres were dissociated to single cells after 7 days by trypsinization and further sieving through 40- µm cell strainers and re-plated at 5×10^3^−10^4^ cells/mL to obtain secondary mammospheres. To form mammo-angiospheres, one part of PKH26^+^BCCs were mixed with 10 parts of GFP^+^E4-ECs (1∶10 ratio) and co-cultured under non-adherent condition for 5–7 days to obtain mammo-angiospheres. Sphere proliferation was measured by the increase in number of mammosphere clusters distinguished by PKH26 staining.

### Flow cytometry and cell sorting

Phycoerythrin (PE) mouse anti-human CD44 (clone G44-26) and Alexa fluor (AF) 647 mouse anti-human CD24 (clone ML5) antibodies were products of BD Biosciences, USA (555479 & 561644, respectively). FcR blocking reagent was from Miltenyi Biotec. (120-000-442). To quantify breast cancer stem cells (BCSCs) in mammospheres, co-cultured and control mammospheres were labeled with AF647-CD24 and PE-CD44. Initially, mammospheres and mammo-angiospheres were trypsinized and strained to obtain single cells. Briefly, cells were resuspended at 1×10^6^ cells/100 µL density in staining buffer containing 5% FBS, 1% BSA, 0.2 mM EDTA in PBS. To enhance the specificity of staining, FcR blocking was added at 5 µL/10^6^ cells prior to incubation with primary antibodies. Primary antibodies were used following the instructions provided by the manufacturer and incubation was done for 1 hour at 4°C. After washing with PBS, the fluorescent light (FL) was quantified using Fluorescence Activated Cell Sorting (FACS) on a SORP FACSAria II (BD Biosciences). PE fluorescent was acquired by 498 nm blue laser and 575/26 nm emission; AF 647 fluorescence (APC) was obtained by 650 nm red laser and 660/20 nm emission; GFP fluorescent was gained by 488 nm blue laser and 510/50 nm emission. Doublets were excluded by FSC-W × FSC-H and SSC-W × SSC-H analysis; single stained channels were used for compensation. Fluorescent minus one was used for gating. Data were processed with FACSDiva 6.3 software (BD Biosciences) or Summit 4.3 (Dako) [15].

Adherent and non-adherent co-cultures of PKH26^+^BCCs and GFP^+^E4-ECs were trypsinized and washed once. BCCs were sorted from ECs based on PE by 498 nm blue laser and 575/26 nm emission and GFP fluorescents by 488 nm blue laser and 510/50 nm emission as described above. Control BCCs were also sorted to normalize sorting stress on all cells.

### Immunofluorescent imaging

In order to study the interaction between PKH26^+^BCCs and GFP^+^E4-ECs in mammo-angiosphere a Leica LSM 700 confocal microscope was used. Spheres were imaged live using glass bottom microwell plates (MatTek Corporation, USA).

### Wound healing and adhesion assays

To evaluate the migratory properties of tumor cells, EC-sorted BCCs were immediately plated at 100% confluence in triplicate in 48-well plates in complete cancer medium. They were grown overnight under normal condition to prevent cell death. The next day, the cells were starved for 6 h (no serum/cytokine) and the wound healing assay was performed. Consequently, the migration capability of cells to close the wound was evaluated after 48 h using ImageJ 64 software.

To assess BCC adhesion capacity, EC-sorted BCCs were plated at a density of 10^4^ cells/well in triplicate in a fibronectin-coated 96-well plate (20 µg/mL) and incubated at 37°C for 60–90 minutes. The adherent cells were fixed in 3.7% paraformaldehyde imaged using an AMG light microscope.

### Real-time PCR (qPCR)

Total RNA was extracted with RNeasy Mini Kit (250) from Qiagen (USA) according to manufacturer's instruction and 1 µg of RNA was used to produce cDNA with the ProtoScript M-MuLV *Taq* RT-PCR kit using the oligo dT primers (New England BioLabs). Quantitative real-time analysis (qPCR) was done with a 7500 Real time PCR System (Applied Biosystems) using a Go*Taq* 2-step RT-qPCR System (Promega, USA) to amplify the gene of interest following the instruction provided. The primer sequences that were used in this study are included in Table S1.

### shRNA transfection

Lentiviral particles containing shRNA against human Jagged1 (sc-37202-V), scrambled lentiviral particles (sc-108080), and polybrene (sc-134220) were purchased from Santa Cruz Biotechnology (USA). In summary, E4-ECs were cultured up to 50% confluence and were then treated with polybrene and lentiviral particles containing shRNA against Jagged1 or scrambled particles. Transfected cells were then selected using puromycin (Sigma, USA), and down-regulation of Jagged1 was assessed by qPCR.

### Xenograft studies

All animal procedures were approved by institutional animal care and use committee (IACUC) of Weill Cornell Medical College. For MDA-231 cell xenografts, 2×10^5^ MDA-231 cells were injected solely, or in 1∶10 mixture with either 2×10^6^ E4-ECs, or E4-EC^Jag1KD^ subcutaneously into NOD.Cg-*Prkdc^scid^ Il2rg^tm1Wjl^*/SzJ (NSG) recipient mice. For 3D sphere xenografts, the mammo-angiospheres were collected and sorted for MDA-231 cells 7 days post co-culture with E4-ECs or E4-ECs^Jag1KD^ for subcutaneous injection in NSG mice. Seven weeks post xenograft injection, tumors were isolated for quantification and imaging. Isolated tumors were immediately embedded in Tissue-Tek embedding media (Sakura, 4583) and were then snap frozen in liquid nitrogen. Consequently, 5- µm sections were prepared and stained with PE-CD31 antibody (BD Biosciences, 560983) to check blood vessel density. Images were taken from tumor foci with Nikon Eclipse TE 2000-U.

### Statistical analysis

All quantitative data are expressed as mean ± standard error of the mean (SEM). Statistical analysis and graphical presentation were performed using SigmaPlot 12 (Systat Software Inc., Chicago, IL) or Excel (Microsoft Corporation). A Shapiro-Wilk normality test, with a p = 0.05 rejection value, was used to test normal distribution of data prior to further analysis. All pairwise multiple comparisons were performed by one-way ANOVA followed by Holm-Sidak posthoc tests for data with normal distribution or by Kruskal-Wallis analysis of variance on ranks followed by Tukey posthoc tests, in case of failed normality test. Paired comparisons were performed by Student's t-tests or by Mann-Whitney rank sum tests in case of unequal variance or failed normality test. Statistical significance was accepted for p<0.05 (*), p<0.01 (**) or p<0.001 (***). All experiments were repeated at least three times.

## Results

### E4-ECs promote BCCs self-renewal, survival, and pro-metastatic properties in a contact-dependent manner

**Figure 1:**
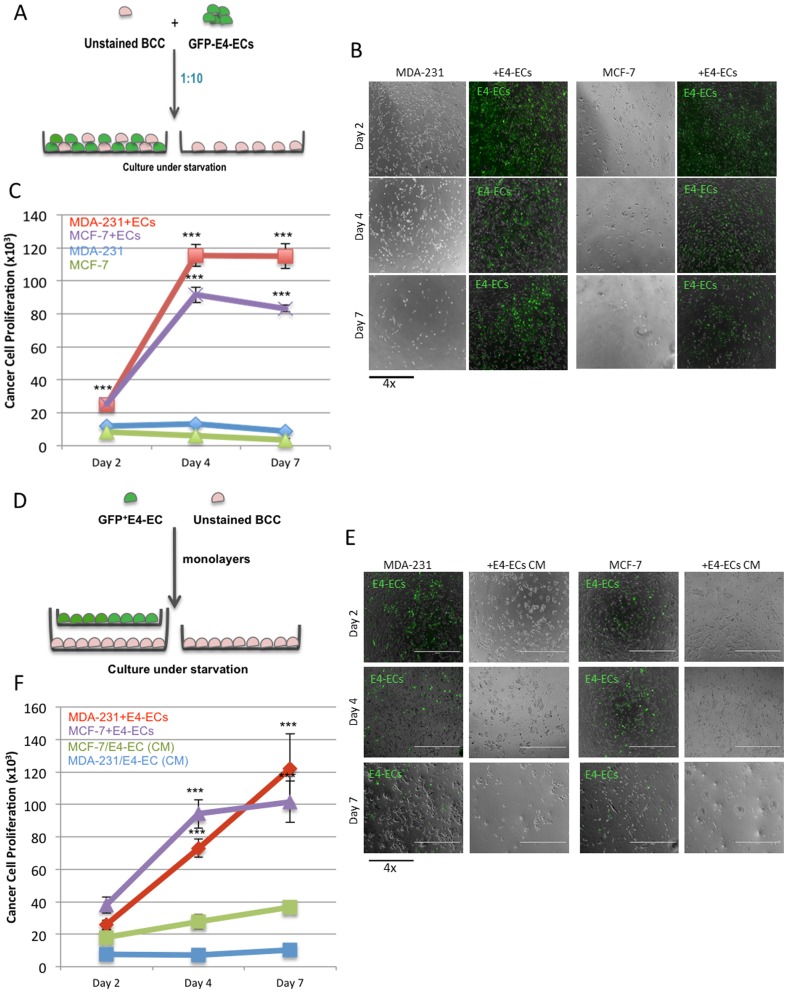
E4-ECs promote BCCs self-renewal and survival in a contact-dependent manner. **A**) Schematic representation of the co-culture system developed for assessing tumor cell proliferation in direct contact with GFP^+^E4-ECs. BCCs and GFP^+^E4-ECs were co-cultivated at 1∶5 ratio without serum and cytokine supplementation and proliferation and survival of BCCs was evaluated 2, 4, and 7 days post co-culture by counting the GFP^-^BCCs using a fluorescent microscope. **B**) Phase contrast and fluorescent microscopy images showing higher BCC proliferation (dark gray cells) in co-culture with GFP^+^E4-ECs as compared with BCCs grown without ECs. **C**) Quantitative analysis of breast cancer cells MDA-231 and MCF-7 proliferation cultured with or without E4-ECs (***p<0.001, mean ± SEM). **D**) Schematic representation of a transwell system used for co-culturing BCCs and E4-EC without any direct contact. BCCs and E4-ECs were separately grown as monolayers in multi-well culture plates and transwell inserts respectively. Then, inserts were positioned in the multi-well plates and both cell types were continued to grow in a serum- and cytokine-free medium (conditioned medium) and the proliferation of BBCs was evaluated at 2, 4, and 7day intervals by manual counting. **E**) Phase contrast and fluorescent microscopy images demonstrating BCCs grown under starvation either alone or in E4-EC conditioned media (CM). **F**) Quantitative analysis of proliferation of breast cancer cells MDA-231 and MCF-7 with or without direct physical contact with E4-ECs demonstrating the importance of contact (***p<0.001, mean ± SEM).

We first investigated if E4-ECs could provide a proliferative niche for cancer cells under complete starvation condition. Using a co-culture system to grow BCCs and E4-ECs jointly in a serum and cytokine-free environment, we showed that BCCs gained a pro-survival advantage through having contact with E4-ECs (Figure 1A–C). MDA-231 and MCF-7 both showed nearly 10-fold increase in their proliferation capacity 4 and 7 days post contact with GFP^+^E4-ECs (Figure 1C). In contrast, BCCs that were grown without GFP^+^E4-ECs demonstrated minimal survival. The proliferative effect could not be reproduced using E4-ECs conditioned media (CM) indicating the importance of cellular contact (Figure 1D).

Early indications on the invasive transformation of tumor cells include their increased migration and adhesion properties. These phenotypic changes are considered as the prerequisites of tumor metastasis. A wound healing assay performed with endothelial-sorted tumor cells (Figure S1A) demonstrated that sorted MDA-231 cells acquired almost 2-fold increase in their ability to close the wound (Figure S1B). The absence of increased migration using E4-ECs conditioned media suggested a contact-dependent mechanism (Figure S1C). Attachment of tumor cells to stromal protein is another indication of tumor aggressiveness. We evaluated the attachment property of tumor cells to fibronectin (FN1) after sorting from GFP^+^E4-ECs. Consistent with increased BCC migration, the attaching capacity of sorted BCCs to matrix improved over 3.5-fold as compared with controls (Figure S1D).

In addition, invasive tumors transform to a more mesenchymal phenotype as a result of down-regulation of their epithelial markers and up-regulation of their mesenchymal features. Consistent with our observation on invasiveness of tumor cells after exposure to ECs, we were able to show the up-regulation of mesenchymal and down-regulation of epithelial markers in MDA-231 cells post sort GFP^+^E4-ECs (Figure S1E). Taken together, our findings suggest a role for ECs in enhancing tumor survival and spreading.

### E4-ECs promote cancer stem cell enrichment

**Figure 2:**
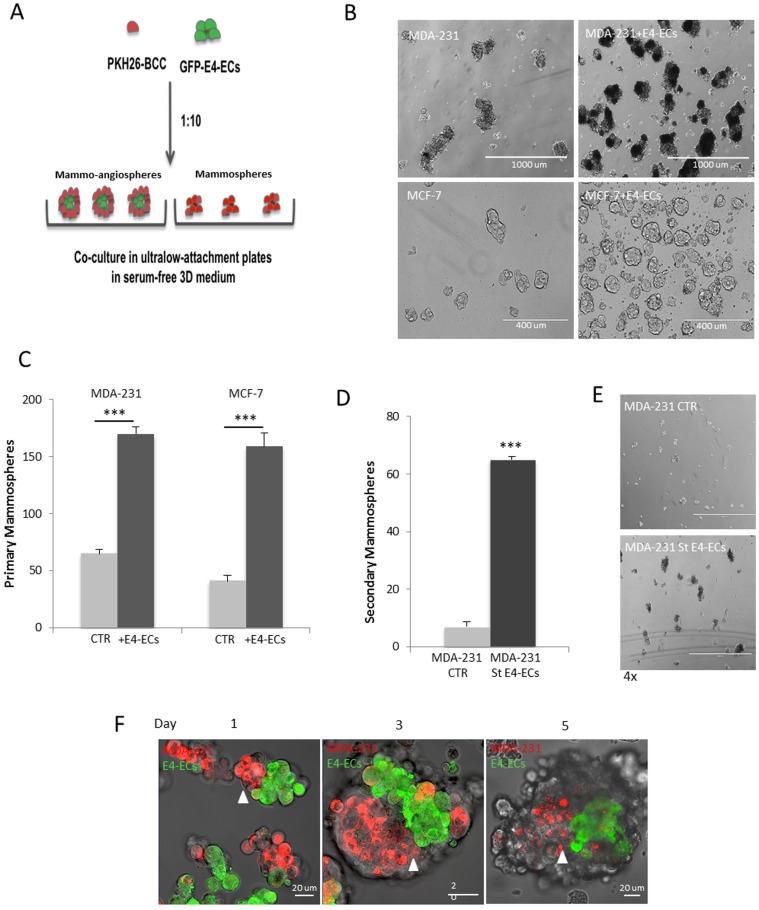
E4-ECs promote mammosphere enrichment. **A**) Schematic representation of sphere forming assay for enriching CSCs. PKH26-BCCs were cultured with and without GFP^+^E4-ECs (1∶10 ratio) under low adherent condition for 5 days and the rate of mammo-angiosphere or mammosphere enrichment was evaluated to determine the role of endothelial cells in CSC propagation. **B**) Phase contrast microscopy images of sphere forming assay illustrating the effect of E4-ECs on mammary stem cell development. **C**) Quantitative analysis of mammosphere formation grown with and without E4-ECs. When E4-ECs mingled with BCCs, the rate of mammosphere formation increased by 3 to 4-fold (***p<0.001, mean ± SEM). **D**) Quantitative analysis of secondary mammosphere formation. Initially, primary mammospheres or mammo-angiospheres were cultivated for 5 days, then dissociated and flow cytometry-sorted mammospheroids were plated under low attachment condition to obtain secondary mammosphere (***p<0.001, mean ± SEM). **E**) Phase contrast microscopy showing significant increase in secondary mammosphere growth in spheroids pre-exposed to E4-ECs. **F**) Immunofluorescent live confocal imaging of the daily process of mammo-angiosphere formation. GFP^+^E4-ECs serve as a core for the accumulation and enrichment of mammary stem cells (red). PKH26^High^ CSCs remain in close vicinity of E4-ECs (white arrowheads).

We applied sphere forming assay to take advantage of anchorage-independence property of cancer stem cells (CSCs) to expand them [27], [28]. In this study, breast cancer stem cells (BCSCs) were enriched following the method described by Dontu et al. resulting in the formation of floating multi-cellular clusters referred to as “*mammospheres*” [27]. When BCCs were mixed and co-cultivated with E4-ECs as described in the method section, it resulted in the formation of structures composed of mammospheres as well as angiospheres (made from E4-ECs). These entities are here referred to as *“mammo-angiospheres”* (Figure 2A).

We initially evaluated the role of tumor endothelium in enhancing BCSCs as mammospheres. A sphere forming assay was performed to evaluate the rate of mammosphere enrichment with and without E4-ECs. We observed significant increase of about 3-fold in mammosphere formation as well as earlier appearance when grown together with E4-ECs (Figure 2B–C). After cell sorting of mammospheres from GFP^+^ angiospheres, an approximately 6-fold increase was quantified in the number of secondary mammospheres produced by sorted tumor cells (Figures 2D&E).

Sphere forming assay has been widely used to enrich mammary stem cells as mammospheres. However, mammary stem cells comprise less than 1% of mammosphere population. Therefore, many investigators have taken advantage of a lipophilic fluorescent dye called PKH for further isolating stem cells from spheres. The staining assay was first utilized by Lanzkron et al. for retrieving hematopoietic stem cells [29]. Later Pece et al. exploited the quiescence nature of stem cells in isolating human normal mammary stem cells by using PKH dye, which tends to retain in slow-dividing cells within a proliferating population [30]. The slow cell division of stem cells also represents an asymmetric cell division that has been described by Pelicci's group in the identification of mammary stem cells stained by PKH dye [31]. Based on these reports, we developed an assay that allowed us to also use PKH26 dye (PE-conjugated) for identification and isolation of slow-dividing, dormant fraction of BCCs with stem/progenitor property. We first looked into the structural integration of PKH26^+^BCCs and GFP^+^E4-ECs by live confocal imaging during 5 days and detected a close interaction between both cell types initiated by formation of an endothelial core at day 1 serving as a scaffold for the further accumulation of tumor spheroids (Figure 2F). Interestingly, the PKH26^High^ mammospheres, which presumably presented the stem cell fraction of mammospheres, could mainly be found in close proximity of GFP^+^E4-ECs (Figure 2F, white arrowheads) that might indicate the critical role of E4-ECs in enhancing and maintaining tumor stemness.

### Functional characterization of E4-EC enriched BCSCs

**Figure 3:**
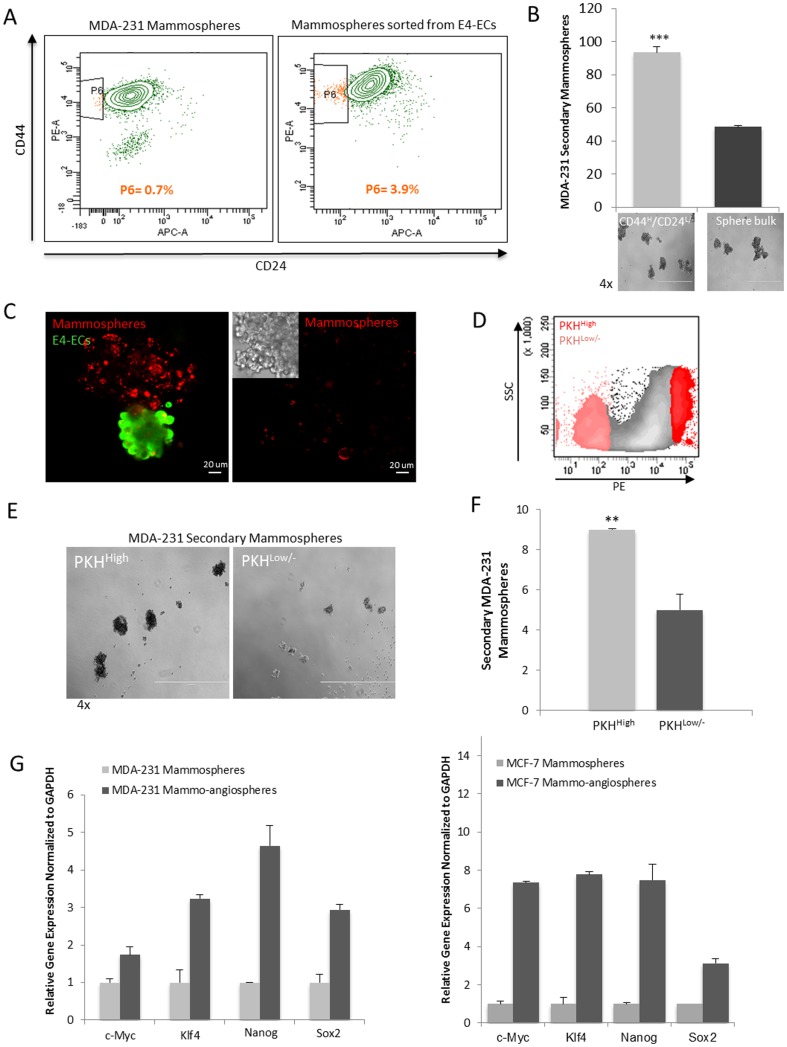
Functional characterization of E4-EC enriched mammospheres. **A**) Flow cytometry analysis of CD44^High^CD24^Low/-^ BCSC population in mammospheres grown with or without GFP^+^E4-ECs. Mammospheres or mammo-angiospheres were dissociated by enzymatic processing and labeled with PE-CD44 and APC-CD24 antibodies after sorting from GFP^+^E4-ECs. Then, the percentage of CD44^High^CD24^Low/-^ mammospheres was evaluated. **B**) Sphere forming assay was performed to compare the stemness capacity of CD44^High^CD24^Low/-^ mammospheres. Primary mammospheres were grown and labeled with PE-CD44 and APC-CD24 antibodies followed by sorting this subpopulation. Consequently, the secondary sphere forming ability of CD44^High^CD24^Low/-^ spheroids was compared with the bulk of primary spheroids (***p<0.001, mean ± SEM). **C**) Confocal images illustrating PKH dye retention in mammospheres cultured with or without E4-ECs. PKH26-stained breast cancer cells (red) while mingled with E4-ECs showed slower cell division and tend to retain the PKH dye. In the absence of E4-ECs, the dye is significantly diluted within the same culture period. **D**) Flow cytometry scatter plot demonstrating gating strategies used to define PKH26^High^ and PKH26^Low/-^ populations for cell sorting. **E**) Phase contrast microscopy of the secondary sphere formation of PKH26^High^ versus PKH26^Low/-^ spheroids. PKH26-stained primary mammo-angiospheres were dissociated and PKH26^High^ and PKH26^Low/-^ populations were sorted and their secondary mammosphere capacity was compared. **F**) Quantification of secondary sphere forming ability of PKH26^High^ versus PKH26^Low/-^ primary spheres demonstrates an almost 2-fold increase in secondary sphere formation by PKH26^High^ cells (**p<0.01, mean ± SEM). **G**) qPCR analysis of the expression of pluripotency markers in primary mammo-angiospheres made by MDA-231 cells (Left panel) or MCF-7 cells (right panel) as compared with mammospheres of each cell type after sorting from GFP^+^E4-ECs.

To further validate the involvement of E4-ECs in enhancing tumor stemness, we applied several approaches. Previous studies identified a subpopulation of CD44^High^CD24^Low/-^ in BCCs that displayed a stem/progenitor cell phenotype in both human tumors and mouse models and were able to form tumors in non-obese diabetic/severe combined immunodeficiency mice [32]. We found that mammospheres that were sorted from angiospheres contained over 5-fold higher percentage of CD44^High^CD24^Low/-^ population compared to mammospheres without E4-ECs (Figure 3A). Consequently, the sorted CD44^High^CD24^Low/-^ population was evaluated for its secondary sphere forming capacity in comparison with the cells from the bulk of mammospheres. Our findings showed that CD44^High^CD24^Low/-^ population was more potent in developing secondary mammospheres indicative of their higher stem/progenitor capacity (Figure 3B).

We also confirmed by immunofluorescence imaging that retention of PKH26 dye in mammospheres was an effect mediated by E4-ECs, as in the absence of ECs PKH26 dye was diluted in dividing mammospheres (Figure 3C). To compare stemness property of PKH26^High^ versus PKH26^Low/-^ spheres, we first sorted mammospheres from angiospheres based on PE^+^/GFP^-^ fluorescents and subsequently determined and sorted the two populations of PKH26^High^ and PKH26^Low/-^ from mammospheres (Figure 3D). Then, sorted PKH26^High^ and PKH26^Low/-^ spheroids were cultured under low adherent condition as described previously and we observed around 2-fold higher secondary mammosphere formation with PKH26^High^ spheres (Figures 3E&F). In addition, the data obtained from q-PCR on key regulators of pluripotency including Sox2, Nanog, Klf4, and c-Myc showed higher expression in angiosphere-sorted mammospheres in comparison to mammospheres with no contact with E4-ECs (Figure 3G). Overall, these observations point out a role for ECs in influencing breast tumor stemness.

### Notch activation mediates E4-ECs induced growth and stemness of BCCs

**Figure 4:**
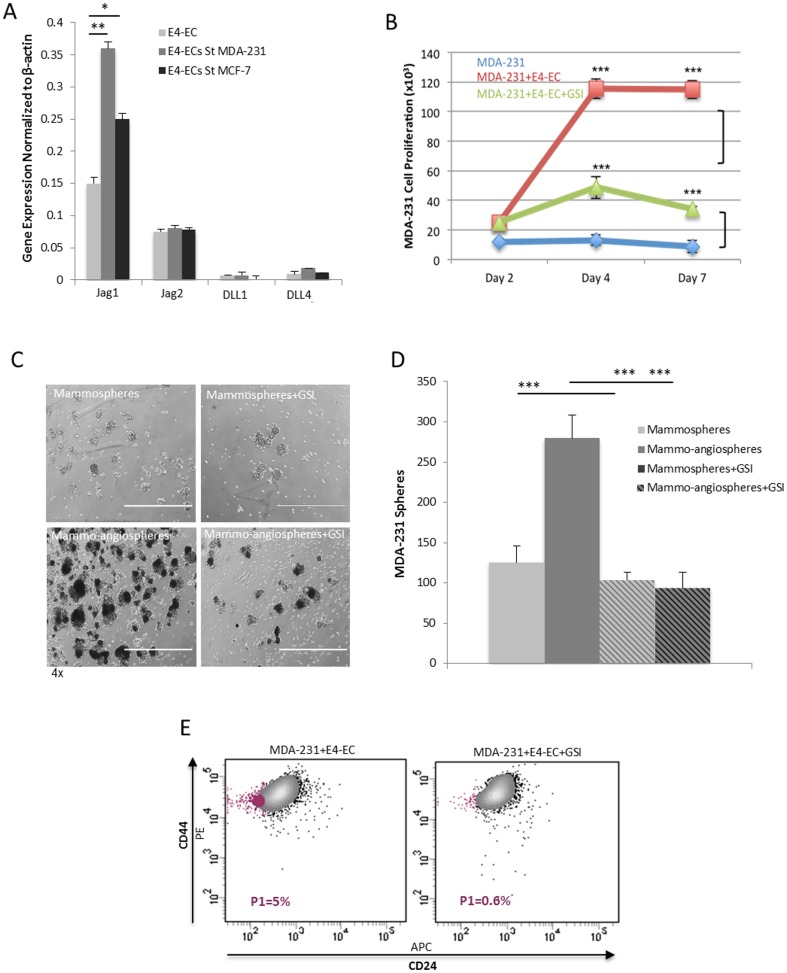
Notch activation mediates E4-ECs induced phenotypic advantages of BCCs. **A**) qPCR analysis showed significant over-expression of notch ligand Jagged1 in E4-ECs after sorting from BCCs MDA-231 and MCF-7 (**p<0.01, mean ± SEM). **B**) Cell proliferation assay performed on MDA-231/E4-ECs co-cultures under complete starvation with or without notch inhibitor, GSI. The results showed dramatic reduction in the ability of E4-ECs to maintain growth and survival of MDA-231 cells when GSI was added (***p<0.001, mean ± SEM). **C**) Mammo-angiosphere forming assay done with or without daily doses of GSI illustrates the effect of notch inhibition on mammosphere enrichment. **D**) Quantitative evaluation of mammosphere formation under GSI treatment. Plot shows that addition of GSI dramatically attenuates E4-ECs role in enhancing mammosphere formation (***p<0.001, mean ± SEM). **E**) Flow cytometry analysis shows significant reduction in CD44^High^CD24^Low/-^ population when mammo-angiospheres were grown under GSI treatment.

We determined the expression of notch ligands such as Jagged1 (Jag1), Jagged2 (Jag2), DLL1, and DLL4 in E4-ECs after contact with MDA-231 and MCF-7 cells. qPCR analysis showed significant up-regulation of Jag1 ligand on E4-ECs after sorting from BCCs (Figure 4A). Also, we observed a dramatic reduction in tumor proliferation and survival when the co-cultures were treated with notch gamma secretase inhibitor (GSI) (Figure 4B). We also showed that notch activation played a role in mammosphere enrichment; once GSI was added to BCC/E4-EC co-cultures, mammosphere growth was reduced by over 3-fold (Figures 4 C&D). Concordantly, growing mammo-angiospheres under GSI treatment resulted in almost 2-fold decrease in CD44^High^CD24^Low/-^ population as was analyzed by flow cytometry (Figure 4E). To rule out the possibility of reduced tumor burden due to increased EC death under GSI treatment, we performed a viability assay 5 days after a sphere forming assay was initiated. We observed comparable survival rate for ECs grown with or without GSI (Figure S2B–C). These findings specify a role for EC Jag1/tumor notch pathway in regulation of tumor growth and stemness.

### Inhibition of endothelial Jagged1 attenuated E4-EC influence on BCCs

**Figure 5:**
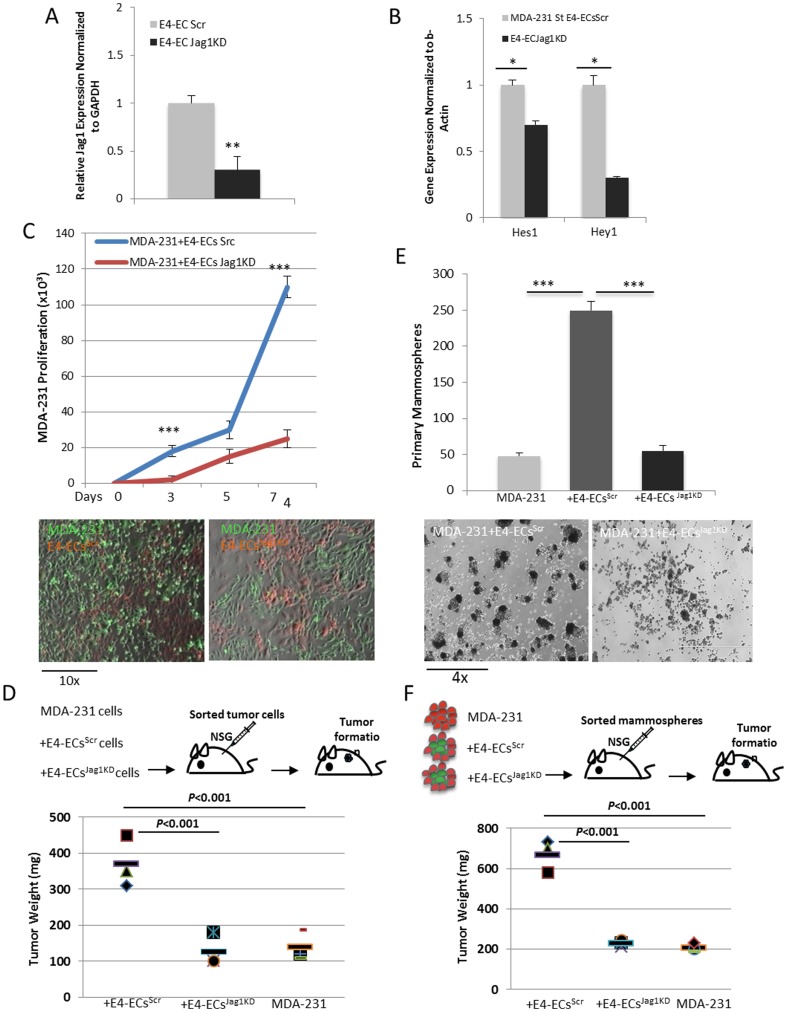
Inhibition of Jagged1 ligand on E4-ECs attenuated their influence on BCCs. **A**) qPCR analysis showing down-regulation of Jagged1 in E4-ECs after transfection with shRNA against Jagged1. **B**) qPCR analysis confirming reduced activation of notch pathway by showing decreased expression of Hes1 and Hey1 notch downstream effector molecules in MDA-231 cells sorted from E4-ECs^Jag1KD^ as compared with MDA-231 cells sorted from E4-ECs^Scr^ (*p<0.05, mean ± SEM). **C**) Cell proliferation assay evaluating the outcome of endothelial Jag1 silencing on BCC self-renewal and survival. Plot represents quantitative analysis of MDA-231 cells grown with either E4-ECs^Scr^ or E4-ECs^Jag1KD^ (upper panel). Fluorescent imaging (bottom panels) illustrates GFP^+^MDA-231 cells in co-culture with mCherry^+^E4-ECs^Scr^ or mCherry^+^E4-ECs^Jag1KD^ (***p<0.001, mean ± SEM). **D**) Schematic representation of subcutaneous injection of MDA-231 with or without E4-ECs^Scr^ or E4-ECs^Jag1KD^ cells (1∶10 ratio) into NSG mice (upper panel). Quantification of tumor weight from NSG mice injected subcutaneously with GFP^+^MDA-231 cells solely or mixed with mCherry^+^E4-ECs or mCherry^+^ECs^Jag1KD^ 7 weeks post injection (bottom panel). **E**) Bar graph representing primary mammo-angiospheres once BCCs were co-cultured with either E4-EC^Scr^ or E4-EC^Jag1KD^ (upper panel). Phase contrast microscopy images (bottom panels) showing mammosphere formation with E4-EC^Scr^ or E4-EC^Jag1KD^ (bottom panel) (***p<0.001, mean ± SEM). **F**) Schematic representation of mammosphere injection into NSG mice (upper panel). Quantification of tumor weight 7 weeks post subcutaneous injection of 1×10^5^ sorted MDA-231 spheroids from primary mammosphere or mammo-angiospheres (bottom panel).

Based on our qPCR data on Figure 4A, Jag1 is over-expressed in E4-ECs that are exposed to BCCs. To confirm if this ligand plays a role in activating tumor notch pathway, we silenced its expression on E4-ECs using shRNA against Jag1. Initially, we determined down-regulation of Jag1 ligand on E4-ECs by qPCR (Figure 5A). Then, E4-ECs^Scr^ and E4-ECs^Jag1KD^ were co-cultured with MDA-231 cells and the expression of canonical notch downstream target genes Hes1 and Hey1 was determined in tumor cells after sorting. qPCR analysis of Hes1 and Hey1 genes confirmed down-regulation of notch signaling pathway in sorted MDA-231 co-cultured with E4-ECs^Jag1KD^ (Figure 5B). Subsequently, the effect of endothelial Jag1 knockdown was determined on proliferation, survival, and enrichment of BCCs and mammospheres. The results demonstrated E4-ECs^Jag1KD^ incompetency to maintain tumor survival under starvation as compared with tumor survival in co-cultures with E4-ECs^Scr^ (Figures 5C, bottom panels). Quantitative analysis showed over 4-fold decrease in tumor cell proliferation when co-cultured with E4-ECs^Jag1KD^ (Figure 5C, top panel). We confirmed that the differences in tumor cell proliferation was not the result of E4-EC accelerated death due to Jag1 silencing by examining DNA synthesis in E4-ECs by EdU incorporation assay and found that DNA synthesis (S phase) decreased in E4-ECs^Jag1KD^ co-cultured with MDA-231 cells (Figure S2A). However, we did not find higher death rate in E4-ECs^Jag1KD^ grown with MDA-231 cells under adherent or low adherent (sphere) conditions, confirming that Jag1 knockdown did not impact death/survival in E4-ECs (Figure S2B–D).

In a classical xenograft assay, we first showed that co-injection of MDA-231 with E4-ECs into NSG mice significantly increased tumor burden, then we confirmed that E4-ECs^Jag1KD^ were not able to promote tumor growth in the xenograft assay (tumor growth was inhibited by 3-fold compared to E4-ECs) (Figure 5D, upper panel, Figure S3A). Confocal images of tumor sections confirmed that difference in tumor burden was not due to aberrant vessel formation and the vessel density of xenograft tumors containing E4-ECs or E4-ECs^Jag1KD^ was not affected (Figure S4). As a control, E4-ECs or E4-ECs^Jag1KD^ injected alone did not form tumor (Data not shown).

To investigate whether the detected BCC proliferation defect *in vitro* and in xenograft also occurred at cancer stem cell level, we performed a sphere forming assay for enriching MDA-231 cells in conjunction with either E4-ECs^Scr^ or E4-ECs^Jag1KD^ and first assessed the rate of mammosphere enrichment *in vitro*. The outcome showed significant reduction in the number of mammospheres when MDA-231 cells were co-cultivated with E4-ECs^Jag1KD^ (Figures 5E, bottom panels). Quantitation of the number of mammospheres showed over 4-fold decrease in mammary stem cell enrichment (Figure 5E, upper panel). To further validate our *in vitro* findings, we sorted MDA-231 spheres from primary mammo-angiospheres and injected them subcutaneously into NSG mice (1×10^5^ cells/mouse) (Figure 5F, upper panel). We detected around 3.2-fold decrease in tumor burden in MDA-231 cells isolated from E4-ECs^Jag1KD^ compared to E4-ECs^Src^ 7 weeks post injection (Figure 5F, bottom panel, Figure S3B). Our *in vivo* findings proved that the effect of the endothelium in xenograft models is concordant with our *in vitro* observations.

## Discussion

In this study, we showed a new role for ECs in enhancing tumor development and progression. Our work demonstrated that ECs Jag1/notch mediated interaction with breast cancer cells increased their tumorigenicity, stemness and invasiveness.

Endothelial cells (ECs) are not merely responsible for provision of oxygen and nutrients; they rather function in many different ways depending on the contexture they reside in. In tumor microenvironment, the crosstalk between ECs and tumor cells was initially shown to activate angiogenesis and vasculogenesis [33]. However, therapeutic agents targeting angiogenesis effector molecules have merely shown transient patient survival resulting in tumor recurrence [34], [35]. These findings indicate alternative roles for ECs in mediating tumor progression. A novel function for ECs was proposed based on studying their role in developmental processes where they induce early embryogenesis through production of specific growth factors [11]–[13], many of which could potentially promote tumor growth. Recently, it has been proposed that ECs regulate tumor growth through the secretion of endothelial-derived paracrine factors referred to as “*angiocrine factors*” [7]. Some angiocrine factors are reportedly implicated in tumorigenesis by promoting tumor angiogenesis [22] while others mediate the perfusion-independent role of ECs in tumor development and metastasis through induction of tumor dormancy, stemness, and epithelial-to-mesenchymal transition [10], [14], [17]. However, the mechanisms by which ECs bestow phenotypic and functional advantages to tumor cells require further investigation.

Our data provided evidence on the significance of cell-to-cell contact between breast tumor and ECs in enhancing tumor growth and development. This observation was in line with previous work by our group on E4-ECs effect on hematopoietic stem cell (HSC) expansion [8], [36]. Our results showed that without contact, E4-ECs were not capable of sustaining BCC survival under starvation. This was consistent with earlier report on the significance of contact between E4-ECs and leukemia cells [23]. Interestingly, the same group has shown that E4-ECs enhanced proliferation of embryonal carcinoma cells without physical contact, suggesting that ECs could support the growth of certain tumor cells in the absence of any physical contact.

The complex process of metastasis is described as a series of events that is referred to as invasion-metastasis cascade [37]. Our preliminary data demonstrated a role for ECs in enhancing tumor migration, adhesion and mesenchymal phenotype. Although these phenotypic changes are implicated as the prerequisite features for local invasion and distant metastasis, the process of endothelial-induced tumor invasion/metastasis needs further investigation by *in vivo* approaches.

Based on recent reports, CSCs are able to actively produce new tumors once inoculated into recipient mice [38], [39]. Also, increasing evidence show that targeting CSC population is challenging due to their characteristics such as quiescence and drug resistance that make them potential cause of tumor relapse and metastasis [40]–[42]. Therefore, targeting this population seems necessary in the treatment of cancer growth and metastasis. Several groups have reported that CSCs are accumulated in the tumor perivascular regions [7], [43] and that the CSCs can be supported by micro-environmental stimuli [44]–[46]. Therefore, looking into the effect of tumor endothelium on CSCs may be beneficial in identification of novel stromal effectors for designing alternative therapeutic methods.

Using several experimental approaches, we have provided strong evidence on the existence and further expansion of CSCs once breast tumor cells are in direct contact with ECs. Here, various markers have been utilized in the detection of CSC population within breast tumor cells, such as sphere forming assay [27], [28], CD44 and CD24 cell surface markers [32], PKH fluorescent dye [31], and expression of pluripotency markers. All methods confirmed the critical role of ECs in enriching mammary stem cells in our setting. In addition, our xenograft assay confirmed the importance of E4-ECs in enhancing tumor initiation potency of mammary stem cells. Recent works have demonstrated a similar role for ECs in improving stemness in head and neck cancer and HSC, respectively [8], [17]. Also, our data identified ECs as potent players in induction of pluripotency markers in mammospheres. The microenvironment-mediated up-regulation of pluripotency markers was previously explained in distinguishing mammary stem cells and as a way of conferring drug resistance to BCCs [47], [48]. However, the possibility of the involvement of vascular endothelium in increasing semness and multi drug resistance through up-regulation of pluripotency markers should be further investigated.

This study highlighted the importance of cell-to-cell contact in ECs interaction with breast tumor cells. In breast cancer notch is primarily activated through up-regulation of its ligands or receptors and are the indicators of poor prognosis [49]. We demonstrated that endothelial Jag1 ligand was overexpressed after direct contact with tumor cells. In agreement with this finding, Butler et al. reported that expression of Jag1 and Jag2 on E4-ECs stimulated the development of HSC [8]. Our *in vivo* findings further determined the role of endothelial Jag1 in enhancing tumor development not only by tumor cells but also by EC-enriched mammary stem cells. A role for notch has been previously implicated in self-renewal and enrichment of normal and cancer stem cells [27], [32], [50]. We demonstrated a role for endothelial Jag1-tumor notch activation in enriching mammary stem cells by several approaches. *In vitro*, we were able to show that under notch inhibition E4-ECs survival was not compromised but influenced BCSC enrichment as was demonstrated by reduced mammosphere formation as well as significant decrease in CD44^High^CD24^Low/-^ subpopulation. Additionally, our *in vivo* results confirmed a role for microenvironment-regulated tumor notch pathway in enhancing the potency of tumor-initiating cells to produce xenografted tumors in nude mice.

In conclusion, we propose an angiogenesis-independent role for ECs in providing a pro-tumoral niche for enhancing cancer proliferation, survival, stemness, and pro-metastatic properties. We have shown that EC effect on cancer development is strongly dependent on direct cell-to-cell contact and is regulated through endothelial Jag1/tumor notch activation. Further elucidation of this mechanism will be beneficial in designing potent stroma-directed therapeutic strategies.

## Supporting Information

Figure S1 E4-ECs enhance the pro-metastatic phenotype of BCCs. **A**) FACS plot shows how GFP^+^E4-ECs and PKH26^+^BCCs populations were gated for sorting. **B**) Wound healing assay performed on sorted MDA-231 cells grown with or without E4-ECs showed that pre-exposure to E4-ECs induced increased migration in MDA-231 cells (***p<0.001, mean ± SEM). **C**) Wound healing assay performed on MDA-231 cells grown in E4-ECs conditioned media (CM) without direct contact showed no significant improvement in MDA-231 cells ability to close the wound. **D**) Cell attachment assay done on MDA-231 (top panels) and MCF-7 (bottom panels) to assess the ability of cells to attach to a substratum (Fibronectin, 20 µg/mL). Counting the attached cells enabled us to estimate over 4-fold increase in the attachment ability of the cells to fibronectin (FN1) when they were pre-exposed to E4-ECs (***p<0.001, mean ± SEM). **E**) qPCR analysis showed the down-regulation of epithelial (E-Cadherin) and up-regulation of mesenchymal (N-Cadherin, FN1, Snail, Vimentin) markers in MDA-231 cells when pre-exposed to E4-ECs.
(TIF)

Figure S2 ECs proliferation and survival is not impacted by GSI treatment or Jag1 silencing. **A**) E4-ECs^Scr^ and E4-ECs^Jag1KD^ were co-cultured with MDA-231 cells and their proliferation rate was measured by EdU incorporation assay after sorting. DNA synthesis (S phase) showed decrease in E4-ECs^Jag1KD^ once they were grown with tumor cells. **B**) Fluorescent microscopy images of mammo-angiosphere growth at day 2 and 5 of culture show no dramatic differences in size and composition of angiospheres when their Jag1 is silenced or grown with GSI. However, mammosphere enrichment was significantly higher when BCCs were mingled with normal E4-ECs and without any GSI treatment (indicated by black arrows). **C**) Quantitative analysis of EC survival rate 5 days after a sphere forming assay was initiated demonstrate no significant difference in the angiosphere growth under Jag1 silencing or GSI treatment. EC death may partly be attributed to the use of serum-free 3D medium for enriching mammo-angiospheres. **D**) Quantitative analysis of EC survival in co-cultures with MDA-231 cells under starvation shows that GSI treatment or Jag1 silencing do not impact EC survival/death rate.
(TIF)

Figure S3 Xenograft tumor formation in NSG mice. **A**) Light microscopy images of MDA-231 xenograft tumors formed in NSG mice. MDA-231 cells were subcutaneously injected alone or in combination with E4-ECs or E4-ECs^Jag1KD^ and tumors were extracted 7 weeks later. **B**) Light microscopy images of xenograft tumors formed in NSG mice after injecting mammospheres dissociated from either E4-ECs or E4-ECs^Jag1KD^.
(TIF)

Figure S4 Confocal image of Xenografted tumors from GFP^+^MDA-231 co-injected with mCherry^+^E4-ECs or mCherry^+^E4-ECs^Jag1KD^. To investigate whether reduced xenograft tumor growth in E4-ECs^Jag1KD^ was the result of defect in their angiogenesis property, we stained xenograft tumor sections with CD31 (vascular marker). Confocal images show that difference in tumor burden was not due to aberrant vessel formation and the vessel density of xenograft tumors containing E4-ECs or E4-ECs^Jag1KD^ was not affected.
(TIF)

Table S1 RT-PCR primers are listed in the table.
(TIF)
