# Supplementary figures and images for: Endothelial Cells Provide a Notch-Dependent Pro-Tumoral Niche for Enhancing Breast Cancer Survival, Stemness and Pro-Metastatic Properties
Source: PLoS One. 2014 Nov 7;9(11):e112424. doi: 10.1371/journal.pone.0112424 (PMC4224483; doi:10.1371/journal.pone.0112424)

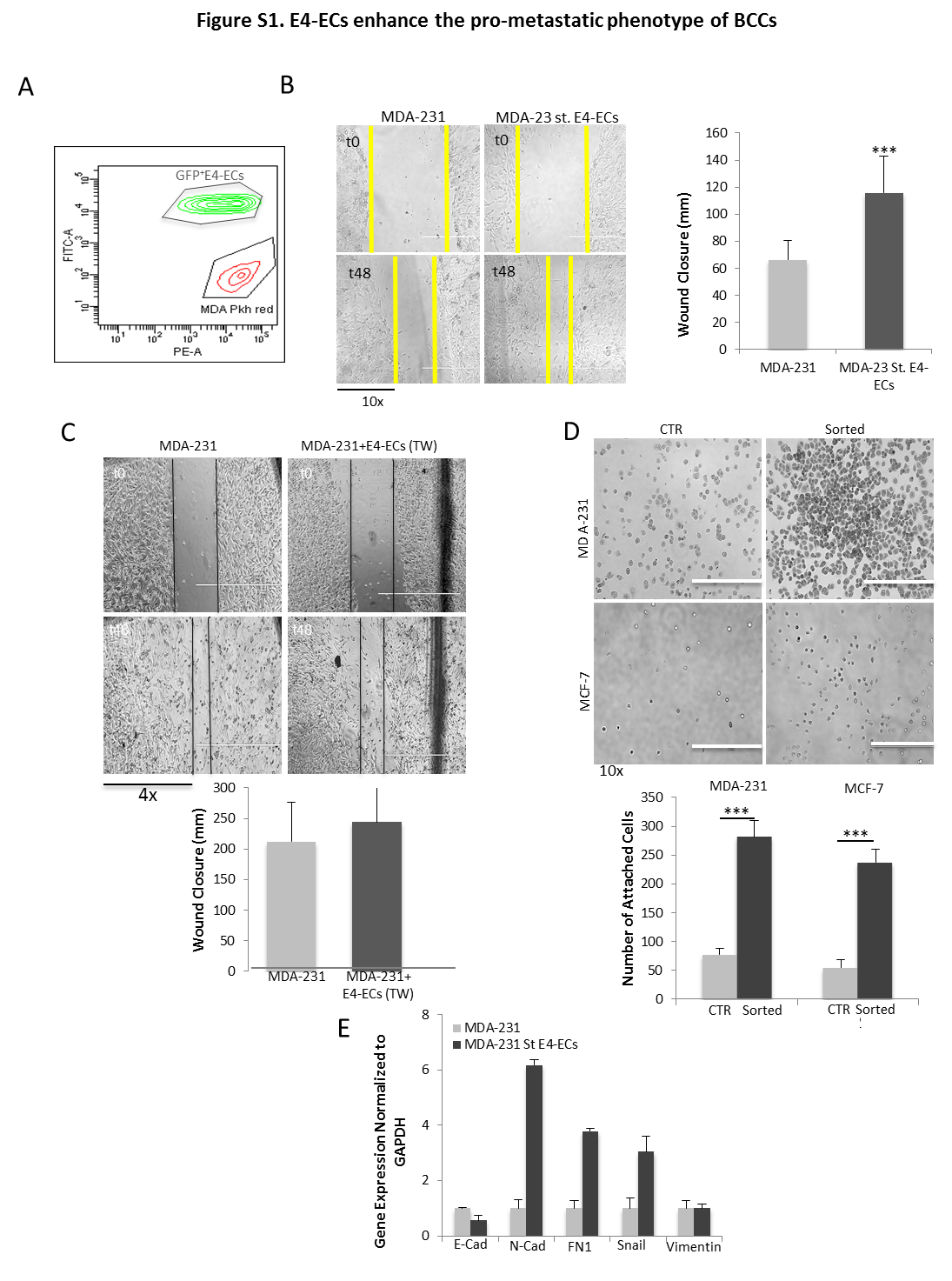

Supplement: Figure S1 — E4-ECs enhance the pro-metastatic phenotype of BCCs. A) FACS plot shows how GFP+E4-ECs and PKH26+BCCs populations were gated for sorting. B) Wound healing assay performed on sorted MDA-231 cells grown with or without E4-ECs showed that pre-exposure to E4-ECs induced increased migration in MDA-231 cells (***p<0.001, mean ± SEM). C) Wound healing assay performed on MDA-231 cells grown in E4-ECs conditioned media (CM) without direct contact showed no significant improvement in MDA-231 cells ability to close the wound. D) Cell attachment assay done on MDA-231 (top panels) and MCF-7 (bottom panels) to assess the ability of cells to attach to a substratum (Fibronectin, 20 µg/mL). Counting the attached cells enabled us to estimate over 4-fold increase in the attachment ability of the cells to fibronectin (FN1) when they were pre-exposed to E4-ECs (***p<0.001, mean ± SEM). E) qPCR analysis showed the down-regulation of epithelial (E-Cadherin) and up-regulation of mesenchymal (N-Cadherin, FN1, Snail, Vimentin) markers in MDA-231 cells when pre-exposed to E4-ECs. (TIF) [file pone.0112424.s001.tif]

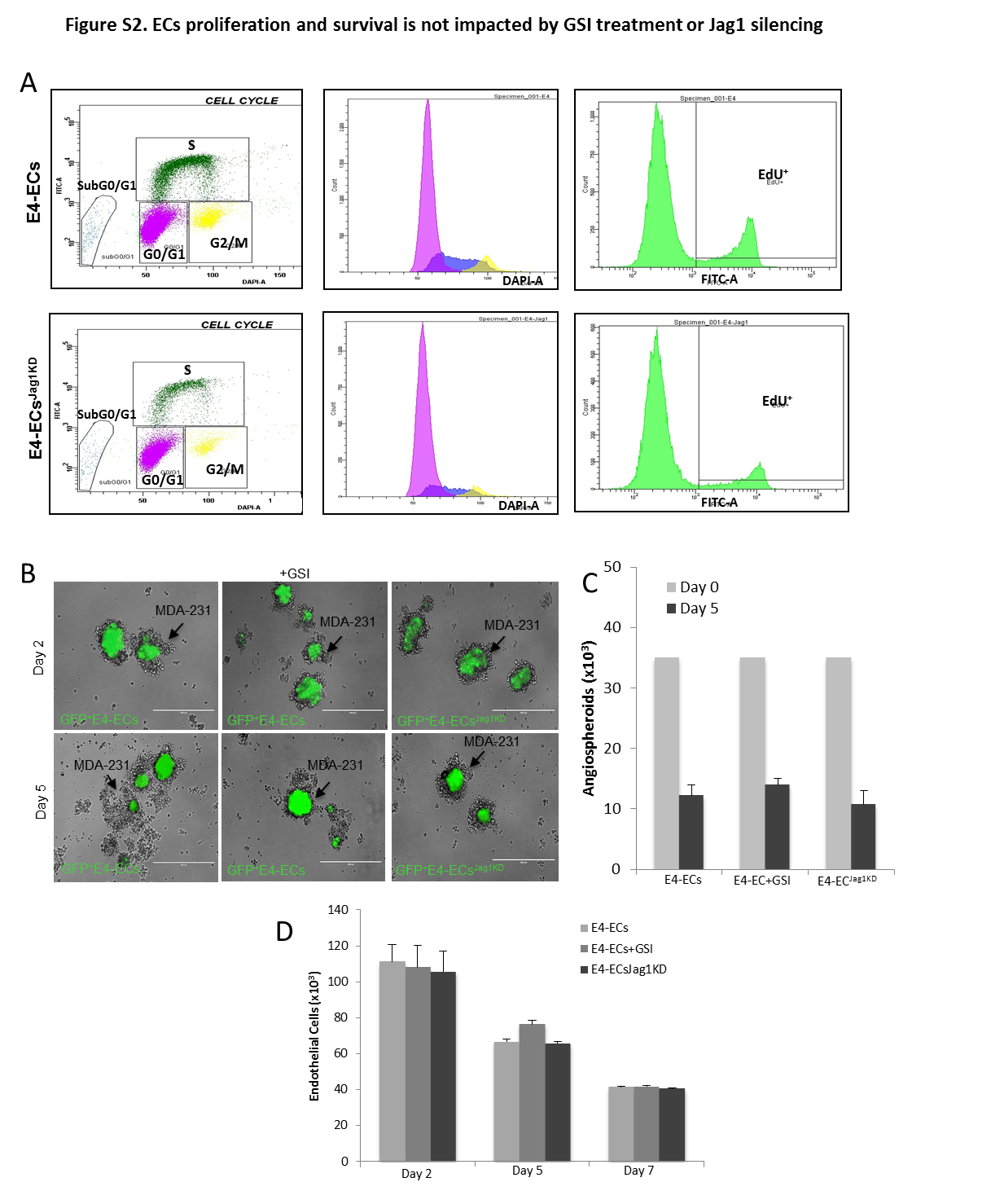

Supplement: Figure S2 — ECs proliferation and survival is not impacted by GSI treatment or Jag1 silencing. A) E4-ECsScr and E4-ECsJag1KD were co-cultured with MDA-231 cells and their proliferation rate was measured by EdU incorporation assay after sorting. DNA synthesis (S phase) showed decrease in E4-ECsJag1KD once they were grown with tumor cells. B) Fluorescent microscopy images of mammo-angiosphere growth at day 2 and 5 of culture show no dramatic differences in size and composition of angiospheres when their Jag1 is silenced or grown with GSI. However, mammosphere enrichment was significantly higher when BCCs were mingled with normal E4-ECs and without any GSI treatment (indicated by black arrows). C) Quantitative analysis of EC survival rate 5 days after a sphere forming assay was initiated demonstrate no significant difference in the angiosphere growth under Jag1 silencing or GSI treatment. EC death may partly be attributed to the use of serum-free 3D medium for enriching mammo-angiospheres. D) Quantitative analysis of EC survival in co-cultures with MDA-231 cells under starvation shows that GSI treatment or Jag1 silencing do not impact EC survival/death rate. (TIF) [file pone.0112424.s002.tif]

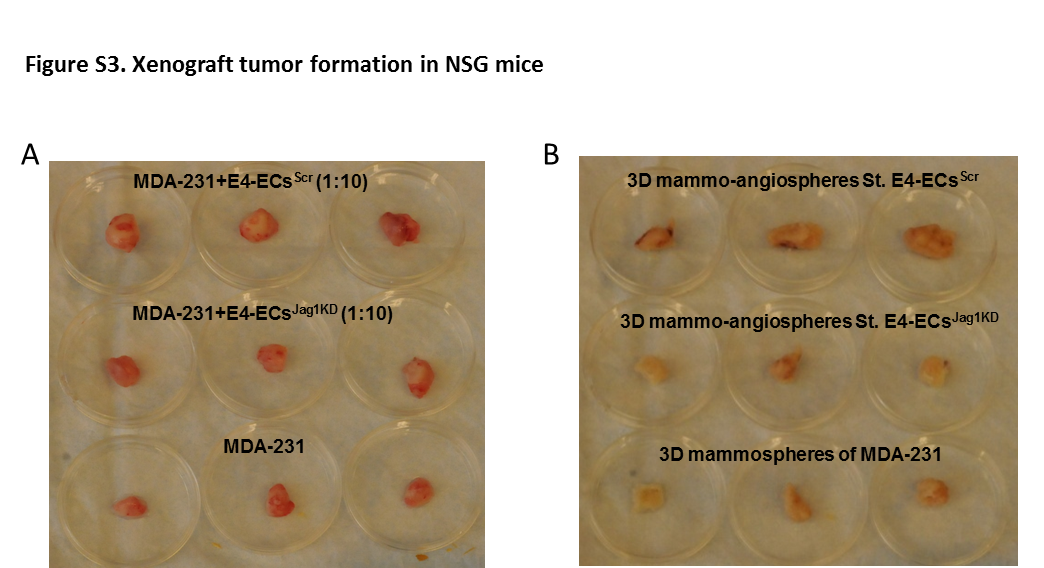

Supplement: Figure S3 — Xenograft tumor formation in NSG mice. A) Light microscopy images of MDA-231 xenograft tumors formed in NSG mice. MDA-231 cells were subcutaneously injected alone or in combination with E4-ECs or E4-ECsJag1KD and tumors were extracted 7 weeks later. B) Light microscopy images of xenograft tumors formed in NSG mice after injecting mammospheres dissociated from either E4-ECs or E4-ECsJag1KD. (TIF) [file pone.0112424.s003.tif]

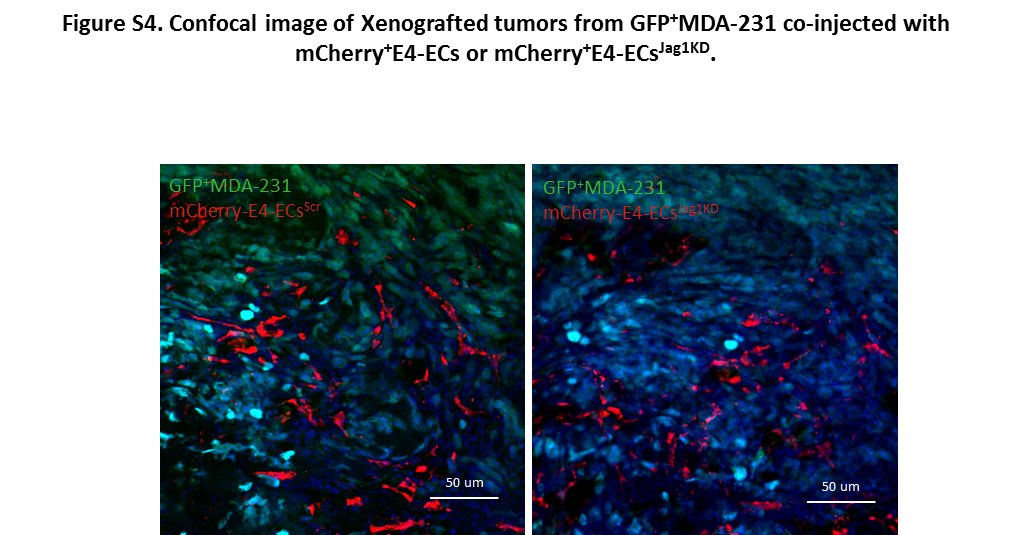

Supplement: Figure S4 — Confocal image of Xenografted tumors from GFP+MDA-231 co-injected with mCherry+E4-ECs or mCherry+E4-ECsJag1KD. To investigate whether reduced xenograft tumor growth in E4-ECsJag1KD was the result of defect in their angiogenesis property, we stained xenograft tumor sections with CD31 (vascular marker). Confocal images show that difference in tumor burden was not due to aberrant vessel formation and the vessel density of xenograft tumors containing E4-ECs or E4-ECsJag1KD was not affected. (TIF) [file pone.0112424.s004.tif]
